# Circulating microRNA biomarkers for lung cancer detection in Western populations

**DOI:** 10.1002/cam4.1782

**Published:** 2018-09-27

**Authors:** Haixin Yu, Zhong Guan, Katarina Cuk, Hermann Brenner, Yan Zhang

**Affiliations:** ^1^ Division of Clinical Epidemiology and Aging Research German Cancer Research Center (DKFZ) Heidelberg Germany; ^2^ Medical Faculty Heidelberg University of Heidelberg Heidelberg Germany; ^3^ German Cancer Consortium (DKTK) German Cancer Research Center (DKFZ) Heidelberg Germany; ^4^ Division of Preventive Oncology German Cancer Research Center (DKFZ) and National Center for Tumor Diseases (NCT) Heidelberg Germany

**Keywords:** early detection, lung cancer, miRNA, Western populations

## Abstract

Lung cancer (LC) is a leading cause of cancer‐related death in the Western world. Patients with LC usually have poor prognosis due to the difficulties in detecting tumors at early stages. Multiple studies have shown that circulating miRNAs might be promising biomarkers for early detection of LC. We aimed to provide an overview of published studies on circulating miRNA markers for early detection of LC and to summarize their diagnostic performance in Western populations. A systematic literature search was performed in PubMed and ISI Web of Knowledge to find relevant studies published up to 11 August 2017. Information on study design, population characteristics, miRNA markers, and diagnostic accuracy (including sensitivity, specificity, and AUC) were independently extracted by two reviewers. Overall, 17 studies evaluating 35 circulating miRNA markers and 19 miRNA panels in serum or plasma were included. The median sensitivity (range) and specificity (range) were, respectively, 78.4% (51.7%‐100%) and 78.7% (42.9%‐93.5%) for individual miRNAs, and 83.0% (64.0%‐100%) and 84.9% (71.0%‐100%) for miRNA panels. Most studies incorporated individual miRNA markers as panels (with 2‐34 markers), with multiple miRNA‐based panels generally outperforming individual markers. Two promising miRNA panels were discovered and verified in prospective cohorts. Of note, both studies exclusively applied miRNA ratios when building up panels. In conclusion, circulating miRNAs may bear potential for noninvasive LC screening, but large studies conducted in screening or longitudinal settings are needed to validate the promising results and optimize the marker panels.

## 1 INTRODUCTION

Lung cancer (LC) is one of the most common forms of cancer and causes of cancer‐related death worldwide. LC was estimated to account for 449 000 cases and 388 000 deaths in Europe, and 214 000 cases and 168 000 deaths in the US in 2012.^1^ The overall 5‐year survival rate of LC is less than 20% as the majority of tumors are diagnosed at late stages, whereas patients with tumors diagnosed at Stage IA have 5‐year survival rates of approximately 70%.^2^ Early detection of malignant tumors could therefore significantly reduce LC mortality. Of the potential screening methods, it has been shown that sputum examinations and chest X‐rays are ineffective in reducing LC mortality.^3^ Low‐dose computed tomography (CT) screening appears to be promising for high‐risk smokers,^4^ but high false‐positive rates, and cost‐effectiveness are still major problems.^3^, ^5^

The possibility of effective noninvasive cancer screening based on molecular markers detected in body fluids, such as microRNAs (miRNAs) in blood, has recently become a major research area.^6^ miRNAs are short (approximately 22 nucleotides in length) non‐coding RNAs that regulate gene expression by affecting the stability and translational rate of their target messenger RNA (mRNA).^7^ Studies showed that circulating miRNAs become dysregulated during tumor development and therefore result in abnormal miRNA profiles in cancer patients.^7^, ^8^ Clinical studies evaluating the diagnostic efficacy of miRNAs in serum/plasma have shed light on the potential of miRNA biomarkers for noninvasive cancer screening, and a number of LC‐related miRNA candidates/panels have already been identified.^9^, ^10^, ^11^

In this review, we provide a systematic and comprehensive summary of the published articles which investigated circulating miRNA candidates for LC detection. We report study characteristics as well as indicators of diagnostic performance of the miRNAs and miRNA panels to provide an overview of where the field stands right now and bring up research questions for future studies. Given the heterogeneity in reported miRNA profiles between ethnicities,^12^ this review focused on studies from Western populations.

## 2 METHODS

The systematic review was conducted according to a predefined protocol. Reporting follows the PRISMA statement.^13^

### 2.1 Literature search

A systematic literature search was performed to identify studies that assessed circulating miRNAs in relation to LC. The PubMed and ISI Web of Science databases were searched for relevant articles that conformed to our inclusion and exclusion criteria and were published up to 11 August 2017. The search was done using the following keyword combinations: ([lung OR pulmonary] AND [cancer OR carcinoma OR neoplasm OR tumor OR adenocarcinoma OR squamous carcinoma OR malignancy] AND [microRNA* OR miRNA* OR miR* OR let‐7*] AND [detection OR diagnosis OR biomarker OR marker] AND [blood OR serum OR plasma]). Duplicate publications were removed.

### 2.2 Eligibility criteria

**Figure 1:**
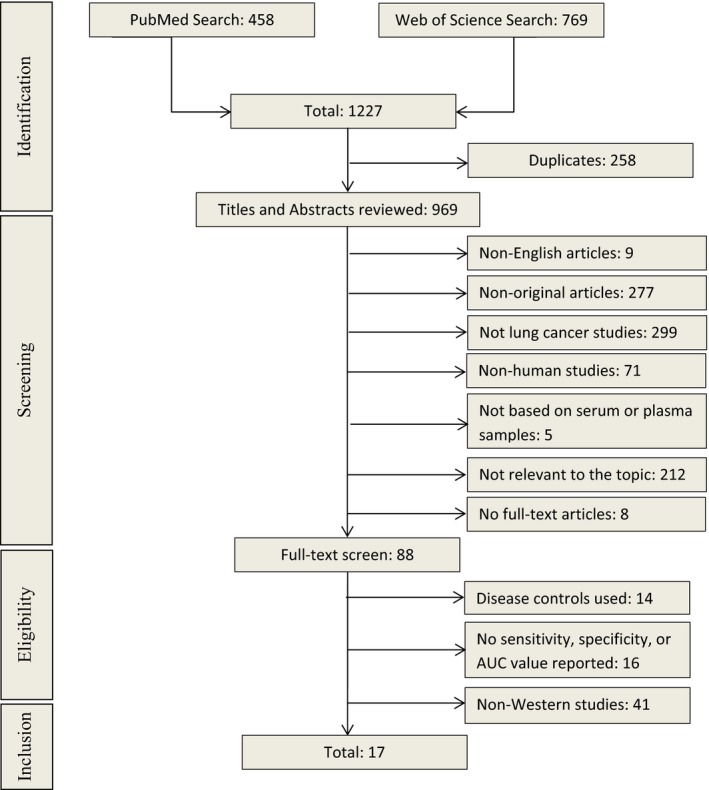
Overview of the literature search process (up to 11th of August 2017)

The initial screening for potential eligible studies was done based upon reading of the title and abstract, and the following exclusion criteria were used (Figure 1): (a) non‐English articles, (b) non‐original articles, (c) not lung cancer studies, (d) non‐human studies, (e) not based on serum or plasma samples, (f) not relevant to the topic, and (g) no full‐text articles. The second round of screening involved reading full‐text articles. At this point, the following studies were excluded: (a) studies using disease controls, (b) studies not reporting critical data regarding diagnostic performance (such as number of cases and controls, sensitivity, specificity, or area under the curve (AUC)), and (c) non‐Western population studies.

### 2.3 Data extraction and statistical analysis

Two reviewers (HY and ZG) independently read and extracted data from the studies that met the inclusion and exclusion criteria described above. Any disagreements were discussed and resolved among the authors. From each study, we extracted available data on first author, publication year, country, study design, basic population characteristics (including size, age, male proportion, histological subtype, and tumor stage for cases), type of bio‐specimen (serum or plasma), miRNA measurement method, targeted miRNA markers, and diagnostic performance indicators (including sensitivity, specificity, AUC, *P*‐value). Individual miRNAs with *P*‐value >0.05 were dropped. Mean or median age and male proportion of included studies were calculated by statistical software R (version 3.3.3) if statistics were not reported but raw data were available. Different miRNA names were incorporated through miRBase database (http://www.mirbase.org/).

### 2.4 Quality assessment

The quality of each included article was assessed according to quality assessment of diagnostic accuracy studies (QUADAS‐2), the most recommended tool for diagnostic accuracy evaluation, and was completed using software Review Manager (version 5.3). Four domains are evaluated for risk of bias in QUADAS‐2: (a) patient selection, (b) index test, (c) reference standard, and (d) flow and timing. The first three domains are also evaluated for applicability concerns.

## 3 RESULTS

### 3.1 Literature search result

A flowchart of the literature search process is given in Figure 1. The initial search yielded 1227 articles using the search terms described above, 458 from PubMed and 769 from Web of Science (Figure 1). Among these, 258 duplicates were removed first. Then, 969 articles went through title and abstract review and a total of 881 articles were excluded according to the above‐mentioned exclusion criteria. The remaining 88 articles were selected for full‐text reading, of which 71 articles were removed: 14 using disease controls, 16 without reporting sensitivity, specificity or AUC values, and 41 reporting in non‐Western countries. In the end, 17 studies evaluating the diagnostic performance of circulating miRNAs in serum or plasma for LC detection published between 2011 and 2017 (Tables 1 and 2) were eligible for this systematic review.^9^, ^10^, ^11^, ^14^, ^15^, ^16^, ^17^, ^18^, ^19^, ^20^, ^21^, ^22^, ^23^, ^24^, ^25^, ^26^, ^27^

**Table 1:** Diagnostic performance of individual miRNA markers in lung cancer in Western populations

Study	Country	Cases vs controls			Specimen	Histology	Stage	miRNA	SEN	SPE	AUC	*P*‐value
		Number	Age (y)	Male (%)								
Zaporozhchenko, 2016^27^	Russia	75/50	65/51	89/84	Plasma	Any LC	IIA‐IV	miR‐19b	69	93	0.81	<0.001
								miR‐21	90	45	0.63	0.022
								miR‐25	56	84	0.66	0.010
								miR‐183	79	71	0.82	<0.001
		53/50	NA/51	NA/84		SCC	IIA‐IV	miR‐19b	75	91	0.83	<0.001
								miR‐25	68	84	0.81	<0.001
								miR‐183	100	43	0.76	0.004
								miR‐205	78	72	0.68	0.048
		18/50	NA/51	NA/84		ADC	IIA‐IV	miR‐19b	58	93	0.77	<0.001
								miR‐21	88	45	0.65	0.041
								miR‐183	100	81	0.92	<0.001
Powrozek, 2016^18^	Poland	90/85	64/57	69/60	Plasma	Any LC	I‐IV	miR‐448	91	75	0.89	<0.0001
								miR‐4478	71	74	0.80	<0.0001
		40/85	NA/57	NA/60		NSCLC	IA‐IIB	miR‐448	85	77	0.89	<0.0001
								miR‐4478	75	68	0.82	<0.0001
Halvorsen, 2016^15^	Norway	100/58	63/58	72/59	Serum	NSCLC	I‐IV	miR‐34b	/	/	**0.62**	0.002^a^
								miR‐125b	/	/	**0.77**	<0.001^a^
								miR‐200b	/	/	**0.83**	<0.001^a^
								miR‐203	/	/	**0.66**	<0.001^a^
								miR‐205	/	/	**0.80**	<0.001^a^
								miR‐429	/	/	**0.79**	<0.001^a^
Chen, 2016^14^	USA	55/53	67/60	56/45	Plasma	NSCLC	I‐II	miR‐21	/	/	0.70	<0.001^a^
								miR‐152	/	/	0.70	<0.001^a^
Wang, 2015^25^^,^ a	USA	108/48	67/59	48/42	Serum	NSCLC	I‐IV	miR‐7	/	/	**0.96**	<0.0001
								miR‐25	/	/	**0.70**	<0.0001
								miR‐193a‐3p	/	/	**0.86**	<0.0001
								miR‐214	/	/	**0.87**	<0.0001
								miR‐483‐5p	/	/	**0.93**	<0.0001
Powrozek, 2015^19^	Poland	90/85	65/57	62/60	Plasma	Any LC	I‐IV	miR‐944	82	91	0.91	<0.0001
								miR‐3662	72	94	0.90	<0.0001
Powrozek, 2015^19^	Poland	NA/85	NA/57	NA/60		SCC	I‐IIIA	miR‐944	86	90	0.93	<0.0001
								miR‐3662	100	56	/	/
		NA/85	NA/57	NA/60		ADC	I‐IIIA	miR‐944	70	80	/	/
								miR‐3662	82	94	0.93	<0.0001
Rani, 2013^20^	Ireland	40/40	66/64	55/55	Serum	ADC	I‐IV	miR‐30c‐1*	/	/	0.74	0.0002
								miR‐146b‐3p	/	/	0.71	0.001
								miR‐550	/	/	0.72	0.0006
								miR‐566	/	/	0.79	0.0001
								miR‐616*	/	/	0.81	0.0001
								miR‐939	/	/	0.82	0.0001
Ma, 2013^17^^,^ c	USA	36/38	67/65	61/66	Plasma	NSCLC	I	miR‐21‐5p	/	/	0.79	0.0013^a^
								miR‐335‐3p	/	/	0.71	0.009^a^
Heegaard, 2012^16^	USA	99/220	NA/68	NA/49	Serum	ADC	IA‐IIB	miR‐146b	/	/	0.55	0.04^a^
Shen, 2011^23^	USA	58/29	68/66	66/66	Plasma	NSCLC	I‐IV	miR‐21	79	66	0.82	0.0002^a^
								miR‐126	69	83	0.76	0.0005^a^
								miR‐182	52	76	0.66	0.0001^a^
								miR‐210	74	69	0.75	0.0003^a^
								miR‐486‐5p	84	69	0.88	0.0006^a^
Roth, 2011^21^	Germany	35/28	55/42	66/NA	Serum	Any LC	I‐IV	miR‐10b	/	/	0.90	0.0001^a^
								miR‐34a	/	/	0.80	0.0001^a^
								miR‐141	/	/	0.88	0.0001^a^
								miR‐155	/	/	0.86	0.0001^a^

ADC, adenocarcinoma; AUC, area under the curve; LC, lung cancer; NA, not available; NSCLC, non‐small‐cell lung cancer; SEN, sensitivity; SPE, specificity; SCC, squamous cell carcinoma.

AUCs in bold fonts represent results from validation set (non‐bold fonts represent results without validation).

*P*‐value represents the difference of miRNA levels between cases and controls (all other *P*‐values represent the statistical significance of AUC values).

a Chinese validation set not included.

miRNAs detected with digital PCR (all other studies detected with qRT‐PCR).

**Table 2:** Diagnostic performance of miRNA panels in lung cancer in Western populations

Study	Country	Cases vs controls			Specimen	Histology	Stage	miRNA	SEN	SPE	AUC
		Number	Age (y)	Male (%)							
Zaporozhchenko, 2016^27^	Russia	75/50	65/51	89/84	Plasma	Any LC	IIA‐IV	‐19b, ‐183	95	95	0.99
Powrozek, 2016^18^	Poland	90/85	64/57	69/60	Plasma	Any LC	I‐IV	‐448, ‐4478	89	79	0.90
		40/85	NA/57	NA/60		NSCLC	IA‐IIB	‐448, ‐4478	90	76	0.90
Halvorsen, 2016^15^	Norway	100/58	63/58	72/59	Serum	NSCLC	I‐IV	Panel A	**88**	**71**	**0.89**
Wozniak, 2015^26^	Russia	100/100	63/60	86/71	Plasma	NSCLC	IA‐IIIA	Panel B	83	80	0.92
		35/100	NA/60	NA/71		ADC	IA‐IIIA		/	/	0.94
		65/100	NA/60	NA/71		SCC	IA‐IIIA		/	/	0.96
Wang, 2015^25^^,^ a	USA	108/48	67/59	48/42	Serum	NSCLC	I‐IV	Panel C	**95**	**84**	**0.95**
Powrozek, 2015^19^	Poland	90/85	65/57	62/60	Plasma	Any LC	I‐IV	‐944, ‐3662	82	92	0.91
		40/85	NA/57	NA/60		NSCLC	I‐IIIA	‐944, ‐3662	92	86	0.88
Sozzi, 2014^25^^,^ b	Italy	69/870	61/56	81/63	Plasma	Any LC	I‐IV	Panel D	**87**	**81**	**/**
Sanfiorenzo, 2013^22^	France	52/20	65/68	75/70	Plasma	NSCLC	IA‐IIIA	Panel E	81	83	0.88
		27/20	NA/68	NA/70		ADC	IA‐IIIA		78	82	0.86
		25/20	NA/68	NA/70		SCC	IA‐IIIA		78	90	0.91
Ma, 2013^17^^,^ ^c^	USA	36/38	67/65	61/66	Plasma	NSCLC	I	‐21‐5p, ‐335‐3p	72	81	0.86
Hennessey, 2012^11^	USA	55/75	68/66	56/67	Serum	NSCLC	I‐IV	‐15b, ‐27b	**100**	**84**	**0.98**
								‐15a, ‐27b	**94**	**75**	**/**
								‐142‐3p, ‐27b	**87**	**76**	**/**
								‐15b, ‐301	**75**	**93**	**/**
								‐27b, ‐301	**75**	**76**	**/**
Heegaard, 2012^16^	USA	220/220	68/68	48/49	Serum	NSCLC	IA‐IIB	Panel F	/	/	0.60
		58/220	NA/68	NA/49		SCC	IA‐IIB	‐221, let‐7a	/	/	0.57
Shen, 2011 23	USA	58/29	69/66	66/66	Plasma	NSCLC	I‐IV	Panel G	86	97	0.93
		24/29	67/66	63/66		SCC	I‐IV		82	97	/
		34/29	68/66	68/66		ADC	I‐IV		92	97	/
Boeri, 2011 10 ^,^ a	Italy	15/54	/	/	Plasma	Any LC	IA‐IV	Panel H	**80**	**90**	**0.85**
		16/54	/	/				Panel I	**75**	**100**	**0.88**
Bianchi, 2011 9	Italy	34/30	62/59	68/67	Serum	NSCLC	I‐IV	Panel J	**71**	**90**	**0.89**
		12/30	64/59	67/67		SCC	I‐IV		**83**	**90**	**0.94**
		22/30	60/59	68/67		ADC	I‐IV		**64**	**90**	**0.85**

ADC, adenocarcinoma; AUC, area under the curve; LC, lung cancer; NA, not available; NSCLC, non‐small‐cell lung cancer; SEN, sensitivity; SCC, squamous cell carcinoma; SPE, specificity.

SENs, SPEs, and AUCs in bold fonts represent results from validation set (non‐bold fonts represent results without validation).

Panel A: ‐429, ‐205, ‐200b, ‐203, ‐125b, ‐34b; Panel B (24 miRs): let‐7c, ‐122, ‐182, ‐193a‐5p, ‐200c, ‐203, ‐218, ‐155, let‐7b, ‐411, ‐450b‐5p, ‐485‐3p, ‐519a, ‐642, ‐517b, ‐520f, ‐206, ‐566, ‐661, ‐340*, ‐1243, ‐720. ‐543, ‐1267; Panel C: ‐214, ‐483‐5p, ‐193a‐3p, ‐25, ‐7; Panel D (24 miRs): ‐101, ‐106a, ‐126, ‐133a, ‐140‐3p, ‐140‐5p, ‐142‐3p, ‐145, ‐148a, ‐15b, ‐16, ‐17, ‐197, ‐19b, ‐21, ‐221, ‐28‐3p, ‐30b, ‐30c, ‐320. ‐451, ‐486‐5p, ‐660. ‐92a; Panel E (11 miRs): ‐155‐5p, ‐20a‐5p, ‐25‐3p, ‐296‐5p, ‐126‐3p, ‐223‐3p, ‐199a‐5p, ‐24‐3p, ‐152‐3p, ‐145‐5p, let‐7f‐5p; Panel F: ‐146b, ‐221, let‐7a, ‐155, ‐17‐5p, ‐29c, ‐27a, ‐106a; Panel G: ‐21, ‐486‐5p, ‐126, ‐210; Panel H (15miRs): ‐92a, ‐30c, ‐30b, ‐28‐3p, ‐19b, ‐15b, ‐142‐3p, ‐140‐5p, ‐106a, ‐660. ‐451, ‐320. ‐221, ‐197, ‐17; Panel I (13 miRs): ‐17, ‐21, ‐451, ‐660. ‐106a, ‐140‐3p, ‐140‐5p, ‐15b, ‐19b, ‐28‐3p, ‐30c, ‐486‐5p, ‐92a; Panel J (34 miRs): ‐92a, ‐486‐5p, ‐484, ‐191, ‐26a, let‐7b, ‐328, ‐30c, ‐342‐3p, ‐30b, ‐26b, ‐142‐3p, ‐331‐3p, ‐103, ‐17, ‐let‐7a, ‐126, ‐22, ‐374a, ‐148b, let‐7d, ‐28‐5p, ‐139‐5p, ‐376a, ‐98, ‐223, ‐142‐5p, ‐140‐5p, ‐29a, ‐148a, ‐133b, ‐32, ‐566, ‐432* (34 miRs).

Chinese validation set not included.

a Prospective case‐control study (all others are cross‐sectional case‐control study).

miRNAs detected with digital PCR (all other studies detected with qRT‐PCR).

### 3.2 Study quality and characteristics

Study quality assessment was completed by two reviewers (HY and ZG) independently. Any initial inconsistencies were resolved by further discussion between the investigators. The vast majority of included studies were of good quality and no high risk of bias or high applicability concerns were found, but there were unclear risk of bias and unclear applicability concerns in patient selection and index test in some studies. The QUADAS‐2 results of the 17 studies are shown in Figures S1 and S2.

Two of the 17 studies are nested case‐control studies,^10^, ^24^ in which incident cases were identified during following up of a prospective cohort, controls were matched disease‐free individuals from the same cohort and blood samples collected at baseline (ie, prior to incidence and diagnosis) were analyzed. The other 15 studies are case‐control studies in which blood samples were taken after cancer diagnosis.^9^, ^11^, ^14^, ^15^, ^16^, ^17^, ^18^, ^19^, ^20^, ^21^, ^22^, ^23^, ^25^, ^26^, ^27^ Of the 17 studies, 11 evaluated individual miRNAs (Table 1), two of which conducted independent validation.^15^, ^25^ Fourteen studies assessed diagnostic performance of miRNA panels (Table 2), six of which carried out independent validation.^9^, ^10^, ^11^, ^15^, ^24^, ^25^ Detailed information on each study, including the number of cases and controls, mean or median age, proportion of males, specimen type, histological subtype, tumor stage, and diagnostic indicators, is summarized in Tables 1 and 2. In addition, Table 1 also shows the *P*‐value for testing the difference of each individual miRNA between cases and controls or the statistical significance of AUC values (indicated in the footnotes of Table 1).

The median (range) of the numbers of cancer cases and controls was 58 (31‐220) and 53 (20‐870), respectively. Seven studies examined miRNAs in serum 9, 11, 15, 16, 20, 21, 25 and 10 in plasma.^10^, ^14^, ^17^, ^18^, ^19^, ^22^, ^23^, ^24^, ^26^, ^27^ Overall, 17 studies evaluating 35 circulating miRNA markers and 19 miRNA panels in serum or plasma were included (total 109 miRNAs). All 17 studies quantified miRNA levels using qRT‐PCR, the most commonly used method for miRNA detection and quantification over the past 5 years. Only one study conducted by Ma et al^17^ additionally used digital PCR to quantify miRNA level. Most of the included studies used individual miRNAs to build up panels, while two studies applied ratios between the expression values of all miRNAs^10^, ^24^ and one study applied differentially expressed miRNA pairs^11^ to build up panels.

### 3.3 Diagnostic performance of miRNA markers

**Figure 2:**
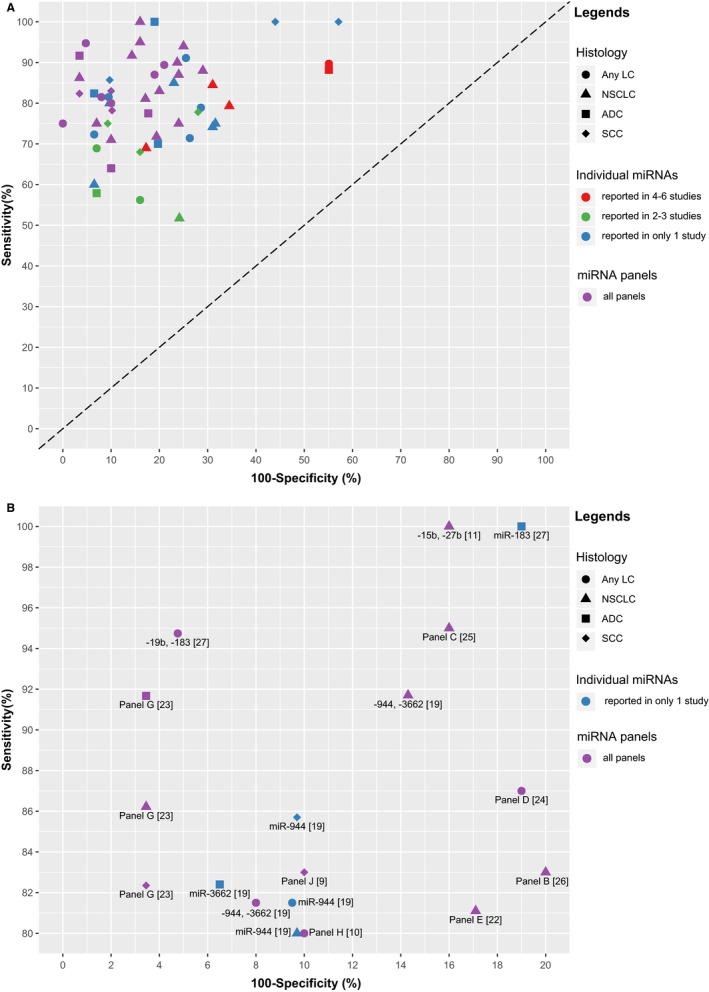
Graphical representation of sensitivity vs specificity of analyzed miRNAs. Sensitivity is plotted on the *y*‐axis while on the *x*‐axis the false‐positive rate is presented (100‐Specificity). A, Overview of all analyzed miRNAs and miRNA panels; B, more detailed representation of miRNAs and miRNA panels with ≥80% sensitivity and ≥80% specificity. The numbers displayed in the brackets represent the corresponding reference numbers. Panel B (24 miRs): let‐7c, ‐122, ‐182, ‐193a‐5p, ‐200c, ‐203, ‐218, ‐155, let‐7b, ‐411, ‐450b‐5p, ‐485‐3p, ‐519a, ‐642, ‐517b, ‐520f, ‐206, ‐566, ‐661, ‐340*, ‐1243, ‐720, ‐543, ‐1267; Panel C: ‐214, ‐483‐5p, ‐193a‐3p, ‐25, ‐7; Panel D (24 miRs): ‐101, ‐106a, ‐126, ‐133a, ‐140‐3p, ‐140‐5p, ‐142‐3p, ‐145, ‐148a, ‐15b, ‐16, ‐17, ‐197, ‐19b, ‐21, ‐221, ‐28‐3p, ‐30b, ‐30c, ‐320, ‐451, ‐486‐5p, ‐660, ‐92a; Panel E (11 miRs): ‐155‐5p, ‐20a‐5p, ‐25‐3p, ‐296‐5p, ‐126‐3p, ‐223‐3p, ‐199a‐5p, ‐24‐3p, ‐152‐3p, ‐145‐5p, let‐7f‐5p; Panel G: ‐21, ‐486‐5p, ‐126, ‐210; Panel H (15 miRs): ‐92a, ‐30c, ‐30b, ‐28‐3p, ‐19b, ‐15b, ‐142‐3p, ‐140‐5p, ‐106a, ‐660, ‐451, ‐320, ‐221, ‐197, ‐17; Panel J (34 miRs): ‐92a, ‐486‐5p, ‐484, ‐191, ‐26a, let‐7b, ‐328, ‐30c, ‐342‐3p, ‐30b, ‐26b, ‐142‐3p, ‐331‐3p, ‐103, ‐17, ‐let‐7a, ‐126, ‐22, ‐374a, ‐148b, let‐7d, ‐28‐5p, ‐139‐5p, ‐376a, ‐98, ‐223, ‐142‐5p, ‐140‐5p, ‐29a, ‐148a, ‐133b, ‐32, ‐566, ‐432*. ADC, adenocarcinoma; LC, lung cancer; NSCLC, non‐small‐cell lung cancer; SCC, squamous cell carcinoma

In total, 109 circulating miRNAs were reported to be statistically significant for LC diagnosis, among which 30 miRNAs were reported in at least two studies (Table 3). Most identified miRNAs were also included in panels, and only nine miRNAs were not part of any panel (Table S1). The smallest panel included only two miRNAs,^11^, ^16^, ^17^, ^18^, ^19^, ^27^ and the largest panel included 34 miRNAs.^9^ An overview of the diagnostic performance of all reported miRNAs and miRNA panels is shown in Figure 2A. For individual miRNAs, the median (range) reported sensitivity and specificity were 78.4% (51.7%‐100%) and 78.7% (42.9‐93.5%), respectively. The median (range) reported sensitivity and specificity of miRNA panels were 83% (64%‐100%) and 84.9% (71%‐100%), respectively. More detailed representation of miRNAs and miRNA panels with ≥80% sensitivity and ≥80% specificity is shown in Figure 2B (three individual miRNAs and 11 miRNA panels). Overall, the diagnostic performance of miRNA panels appears better than that of individual miRNAs.

**Table 3:** Summary of studies reporting significant associations of miRNAs with lung cancer in Western populations (only miRNAs that have been reported in ≥2 studies)

miRNA	Zaporozhchenko, 2016 27	Halvorsen, 2016 15	Chen, 2016 14	Wozniak, 2015 26	Wang, 2015 25	Sozzi, 2014 24	Sanfiorenzo, 2013 22	Rani, 2013 20	Ma, 2013 17	Hennessey, 2012 11	Heegaard, 2012 16	Shen, 2011 23	Roth, 2011 21	Boeri, 2011 10	Bianchi, 2011 9	Number of studies
miR‐21	△↑		△↑			○			△↑			○↑		○		6
miR‐155				○↑			○↑				○↓		△↑			4
miR‐126						○	○↓					○↓			○	4
miR‐486						○						○↓		○	○	4
miR‐17						○					○↓			○	○	4
miR‐142‐3p						○				○				○	○	4
miR‐25	△↓				○↑		○↑									3
miR‐15b						○				○				○		3
miR‐19b	○					○								○		3
miR‐221						○					○↓			○		3
miR‐30c						○								○	○	3
miR‐92a						○								○	○	3
miR‐106a						○					○↓			○		3
miR‐140‐5p						○								○	○	3
miR‐30b						○								○	○	3
miR‐566				○↓				△↑							○	3
miR‐145						○	○↓									2
miR‐182				○↓								△↑				2
miR‐223							○↑								○	2
miR‐148a						○									○	2
miR‐197						○								○		2
miR‐205	△‐	○↑														2
miR‐28‐3p						○								○		2
miR‐320						○								○		2
miR‐451						○								○		2
let‐7a											○↓				○	2
let‐7b				○↑											○	2
miR‐140‐3p						○								○		2
miR‐203		○↑		○↓												2
miR‐660						○								○		2

○ represents miRNAs which are part of a panel; △ represents miRNAs which have only been analyzed individually and not as a part of a miRNA panel; ↑ represents upregulation; ↓ represents downregulation; and ‐ represents no difference in overall study population.

Six of 17 studies recruited LC cases of any histological subtypes,^10^, ^18^, ^19^, ^21^, ^24^, ^27^ 10 studies recruited only non‐small‐cell lung cancer (NSCLC) patients,^9^, ^11^, ^14^, ^15^, ^16^, ^17^, ^22^, ^23^, ^25^, ^26^ and only one study specifically assessed adenocarcinoma LC cases (ADC).^20^ For subgroup analysis, seven studies performed histology‐specific analysis (Tables 1 and 2),^9^, ^16^, ^19^, ^22^, ^23^, ^26^, ^27^ and five studies performed stage‐specific analysis (Table S2).^9^, ^15^, ^18^, ^23^, ^26^ In histology‐specific analyses, several studies observed differential sensitivity, specificity, or AUC values in different histological subtypes, ADC and squamous cell carcinoma (SCC), for the same miRNA or miRNA panel.^9^, ^19^, ^22^, ^23^, ^26^, ^27^ This indicates that miRNAs might play different roles in different histological subtypes of LC, but no histology‐specific miRNA could be identified as the diagnostic performance of miRNAs showed limited differences between different histological subtypes (Table 1). In stage‐specific analyses, several studies showed that diagnostic efficacy of either miRNAs or miRNA panels in advanced stage of LC seems to be better than in early stage of LC; however, the differences with respect to AUC were rather small (Table S2).

Among the 17 studies, two studies evaluated miRNA panels in a prospective setting. Boeri et al^10^ derived and verified a panel of 15 miRNAs for predicting LC incidence in 2 years in a computed tomography (CT) screening trial and yielded sensitivity and specificity of 80% and 90%, respectively. In independent samples of the same trial, Sozzi et al^24^ validated a panel of 24 miRNAs that consisted of the 15 miRNAs in Boeri's study's panel 10 and extra nine miRNAs also identified by Boeri's study,^10^ which showed sensitivity and specificity of 87% and 81%, respectively. In both studies, the algorithm for building up panels was based on miRNA ratios, which were computed between all investigated miRNAs that were consistently expressed in plasma. Boeri et al^10^ suggested that the “ratio method” has equal robustness as the common miRNA normalization but can reduce potential bias introduced by common normalization methods.

There were 30 miRNAs reported at least twice, among which miRNA‐21 was the most frequently reported (six studies), followed by miR‐155, miRNA‐126, miRNA‐486, miRNA‐17, and miRNA‐142‐3p (all four studies) (Table 3). However, higher frequency of reports did not automatically entail the best diagnostic efficacy. For example, the median sensitivity of miRNA‐21 was 88.2% (79.3%‐89.7%), but its median specificity was relatively low, only 44.9% (44.9%‐65.5%).

### 3.4 Direction of dysregulation of circulating miRNAs

Of the 17 studies, 13 studies described the direction of dysregulation of miRNAs in blood, and four studies had no information about miRNA dysregulation (Table S1). Among the 30 miRNAs reported in at least two studies, the overall dysregulation direction of different miRNAs was not always consistent, that is, for six miRNAs, contradictory results were described (Table 3). However, several miRNAs, such as miR‐21 and miR‐126, were consistently reported to have the same dysregulation direction in every corresponding study regardless of histological subtype, stage, or sample type.^14^, ^17^, ^22^, ^23^, ^27^

Some miRNAs displayed no significant differences between overall LC cases of any histological subtype and controls in several studies, but they showed differential expression between LC cases of specific histological subtype and controls, and a few of them were even included in miRNA panels (Table S1). For example, in Zaporozhchenko's study,^27^ miR‐205 levels showed no significant difference between any LC cases and controls, but it was significantly lower in SCC cases compared with controls. Furthermore, in Wozniak's study,^26^ let‐7c, miR‐1267, miR‐206, miR‐519a, miR‐520f, miR‐543, and miR‐720 alone showed no significant difference between NSCLC cases and controls; nonetheless, these miRNAs were incorporated into a 24‐miRNA panel and contributed to generate an AUC value of 0.92.

## 4 DISCUSSION

In this systematic literature review, we identified 17 studies evaluating the diagnostic performance of serum and plasma miRNA markers for LC detection in Western populations. A total number of 109 circulating miRNAs were suggested to hold potential for detection of LC. Most studies incorporated individual miRNA markers as panels (with 2‐34 markers), and multiple miRNA‐based panels generally outperformed individual markers. Two promising miRNA panels were discovered and verified in prospective cohorts.^10^, ^24^ Of note, both of these studies exclusively applied miRNA ratios when building up panels. Histology‐ and stage‐specific diagnostic performances were also explored by small number of studies; however, differences with respect to AUCs were very limited.

Overall, the diagnostic performance of the investigated circulating miRNAs and miRNA panels for LC detection appears to be rather promising, with the sum of sensitivity and specificity by far exceeding 100% in most cases (Figure 2A). There were even three individual miRNAs and 11 miRNA panels with both sensitivity and specificity above 80% (Figure 2B). Some miRNA panels even showed very good diagnostic performance. For example, Zaporozhchenko et al^27^ used a panel composed of miR‐19b and miR‐183 in plasma to detect any histological subtype of LC, and the reported sensitivity and specificity reached 95% and 95%, respectively. Also, Shen et al^23^ used plasma miR‐21, miR‐486‐5p, miR‐126, and miR‐210 to form a panel for the detection of lung adenocarcinoma which yielded 92% sensitivity and 97% specificity. Some miRNA panels even seemed to be useful for prediction of LC incidence 1‐2 years prior to diagnosis in high‐risk populations, with sensitivity and specificity both over 80%.^10^, ^24^ However, most of the included studies were case‐control studies with blood sampling after diagnosis of cases and the sample sizes were relatively small. Most importantly, however, many of the seemingly most promising markers and panels were not independently validated, and reported indicators of diagnostic performance may be overoptimistic. Future validation is therefore indispensable. Such validation should preferably be done within the context of prospective cohort studies.

Although the origin of miRNAs in blood and other body fluids is not fully elucidated yet, miRNAs have specific profiles in different diseases and pathological processes and have shown great potential in the diagnosis and prognosis of various diseases in addition to LC, such as other common cancers, inflammation, and autoimmune diseases.^28^, ^29^, ^30^, ^31^, ^32^, ^33^ With the increasing number of miRNAs with reported association with LC, the low degree of overlap of lung cancer‐specific miRNAs among different studies has become a major concern in applying miRNA for LC detection.^6^ Among the 109 miRNAs included in this review, only 30 miRNA were reported in at least two studies and unlike miR‐21 which consistently demonstrated increased levels in cancer patients, there were miRNAs with reported opposite expression patterns, such as miR‐155, miR‐182, miR‐203, miR‐205, miR‐25, and miR‐566 (Table S3), despite some of them showing good diagnostic performance for LC detection.

One of the causes for the heterogeneity of reported miRNA biomarkers is the differences in study populations. Different tumor histological subtypes or stages of LC cases seem to display at least partially varying miRNA profiles in blood,^9^, ^15^, ^18^, ^19^, ^22^, ^23^, ^26^, ^27^ which is possibly due to different roles some miRNAs can play in tumor initiation and progression in histologically distinct tumor microenvironments. For example, at defined levels of specificity, sensitivities tentatively increasing with progression of cancer stages were observed for a 34‐miRNA panel in Bianchi et al's study^9^ (sensitivities of 59% and 92%, respectively, for Stages I and II‐IV, at specificity of 90%), and for a 4‐miRNA panel in Shen et al's study^23^ (sensitivities of 73%, 87%, 92%, and 94%, respectively, for Stages I, II, III, and IV, at specificity of 97%,). However, stage‐specific analyses of included studies were based on very limited number of cases. In addition, studies showed that also other population characteristics such as age, weight, smoking status, and ethnicity can affect the identification of miRNA markers.^12^, ^34^, ^35^, ^36^ Even other benign diseases such as chronic obstructive pulmonary disease (COPD), asthma, and tuberculosis can alter blood miRNA profiles and make some study participants unsuitable controls.^37^, ^38^, ^39^ Therefore, in this systematic review, we selected Western populations and excluded studies with disease controls to reduce the heterogeneity of included miRNAs caused by above‐mentioned factors.

Sample preparation is an essential pre‐analytical factor affecting the identification of potential marker candidates. Since the concentrations of cellular miRNAs are relatively high compared to those in plasma and serum, a second high‐speed centrifugation or filtration step during blood processing is recommended.^40^, ^41^, ^42^, ^43^ This would serve to remove the potentially retained cells and cell debris from the plasma or serum fraction to minimize the possibility of blood cell contamination of the samples which could lead to an erroneous interpretation of the results. However, only few of the included studies applied such a high‐speed centrifugation step (Table S4). Hemolysis of samples is another factor that can cause variability in miRNA findings.^44^ Erythroid‐specific miRNAs, such as miR‐15b, miR‐16, miR‐141, miR‐451, and miR‐486, are proposed indicators of hemolysis, and their levels can increase up to 50‐fold in hemolyzed samples.^41^, ^42^, ^45^, ^46^ Of the included 17 studies, only three studies^22^, ^24^, ^25^ reported having taken sample hemolysis into account during data processing.

Although both plasma and serum are acceptable sample types for the analysis of circulating miRNAs and high correlation of miRNA concentrations between plasma and serum has been observed,^47^ there are differences between the miRNA profiles obtained from different sample types, which may account for the heterogeneity of reported miRNA biomarkers between studies using plasma samples and those using serum samples. Compared to plasma samples, miRNAs in serum samples have been reported to be higher in concentration but smaller in diversity, suggesting that the coagulation process may affect the amount and species of circulating miRNA.^47^, ^48^ In addition, hemolysis is more likely to affect plasma miRNA profiles during sample preparation,^41^, ^46^ and certain types of anticoagulants used in plasma, such as heparin and EDTA, could also influence the abundance of miRNAs quantified by qPCR.^49^, ^50^

Differences in miRNA extraction and quantification methods as analytical factor could also affect the identification of cancer‐specific miRNAs. Studies indicated that the miRNeasy kit had better miRNA extraction efficiency compared with other miRNA extraction kits.^51^, ^52^ Still, the extraction methods in the included studies were diverse and only few studies used the miRNeasy kit (Table S4). Over the past years, quantitative real‐time polymerase chain reaction (qRT‐PCR) has become the most commonly used method for miRNA detection and all the included studies applied it. Notably, Ma et al^17^ additionally used digital PCR and found that it had a higher sensitivity to detect miRNA copy numbers compared to qRT‐PCR. New and constantly improving technologies, such as next‐generation sequencing (NGS), might also offer a feasible alternative to real‐time PCR‐based methods and enable the detection of novel miRNAs as well as a larger number of miRNA targets per sample in the future.^53^

Another important yet unresolved issue present in circulating miRNA investigation is normalization. At present, no circulating miRNAs have been established as suitable endogenous controls for normalization in plasma or serum. Some researchers even use circulating miR‐16 for this purpose (Table S4), despite its high variability or altered expression in the circulation of cancer patients as well as in hemolyzed samples.^8^, ^41^, ^54^ However, there are some approaches which can be used to minimize experimental variation, such as spiking‐in synthetic miRNAs from another species (*C. elegans* or *A. thaliana*) to check for technical variability during miRNA extraction and processing the same or using constant volumes of samples at each step of the experimental process to somewhat standardize the RNA input.^55^, ^56^

Considering the limitations mentioned above, several studies tried to develop new bioinformatics tools to reduce the analysis bias.^10^, ^11^, ^24^ For example, Hennessey et al^11^ introduced differentially expressed miRNA pairs in serum for NSCLC diagnosis. The differentially expressed miRNA pair of miR‐15b and miR‐27b yielded 100% sensitivity and 84% specificity for distinguishing NSCLC cases and healthy subjects. In another example, Boeri et al^10^ used miRNA ratios instead of just quantities of individual miRNAs in plasma as markers in their nested case‐control study. These miRNA ratios showed a good predictive value for LC development in the next 1‐2 years in a high‐risk smoking population with sensitivity and specificity of 87% and 81%, respectively.

Diagnostic or predictive accuracy of miRNAs is usually enhanced by combination of multiple individual miRNAs as a panel. Currently most studies build up panels based on miRNAs that showed statistically significant associations with LC. This may only capture the main effects of the included miRNA markers. However, it is known that miRNAs can interact with one another,^57^ which may also contribute to the diagnosis or prediction of the disease, as exemplified by some studies^26^, ^27^ in which miRNAs that were not differentially expressed in individual analysis made up components of miRNA panels. Future studies should thus optimize the marker selection procedure by modeling both the main and the interacting effects of the miRNA markers.

## 5 CONCLUSION

Our review suggests that circulating miRNAs have great potential to be used as markers for LC detection and may be promising candidates for general cancer screening. Compared to previous reviews,^39^, ^58^, ^59^, ^60^, ^61^, ^62^, ^63^, ^64^, ^65^ we employed a broader inclusion criterion by including all histological types of LC cases, and we focused on studies conducted in Western populations in order to reduce a primary source of heterogeneity in miRNAs profiles. Although previous reviews have reported tremendous heterogeneity in included studies and inconsistency in LC‐related miRNA markers, very few reviews explored the sources of the heterogeneity.^62^, ^65^ We comprehensively addressed heterogeneity from multiple perspectives, including study populations, biological sample types and processing, methodology in miRNA detection, and data normalization and analysis. Thorough attention to those factors may help to standardize miRNA analytical procedures in the future. In particular, the following implementations may help to reduce measurement and analytical bias and to improve diagnostic performance: minimization of pre‐analytical or analytical variability, utilization of larger prospective studies, improvement of miRNA detection technologies, and development of new analysis methods. Another important step toward the translation of these findings into clinical practice and routine is the selection and validation of truly relevant circulating miRNAs for the formation of diagnostically superior miRNA panels or even multi‐marker combinations with other types of biomarkers. It should be noted, however, that the reported miRNA markers in this review were all derived from the Western populations to take into account heterogeneity of miRNA profiles between ethnicities. Generalization of these miRNA markers thus should be carried out with caution, as the findings summarized in this systematic review may not apply to non‐Western populations.

## CONFLICT OF INTERESTS

The authors declare that they have no competing interests.

## AVAILABILITY OF DATA AND MATERIAL

All data generated or analyzed during this study are included in this published article and its supplementary information files.
