# Supplementary material for: Circulating microRNA biomarkers for lung cancer detection in Western populations
Source: Cancer Med. 2018 Sep 27;7(10):4849–62. doi: 10.1002/cam4.1782 (PMC6198213; doi:10.1002/cam4.1782)
Supplement: Supplementary file 1 [file CAM4-7-4849-s001.doc]

**Supplemental Material**

**Circulating MicroRNA Biomarkers for Lung Cancer Detection in Western Populations**

Haixin Yu^1, 2^, Zhong Guan^1, 2^, Katarina Cuk^1^, Hermann Brenner^1, 3, 4^, Yan Zhang^1^

^1^Division of Clinical Epidemiology and Aging Research, German Cancer Research Center (DKFZ), Im Neuenheimer Feld 581, 69120 Heidelberg, Germany

^2^Medical Faculty Heidelberg, University of Heidelberg, Heidelberg, Germany

^3^German Cancer Consortium (DKTK), German Cancer Research Center (DKFZ), Heidelberg, Germany

^4^Division of Preventive Oncology, German Cancer Research Center (DKFZ) and National Center for Tumor Diseases (NCT), Heidelberg, Germany

**Table of Contents**

**Table S1, pages 3-5**

**Table S2, page 6**

**Table S3, page 7**

**Table S4, page 8**

**Figure S1, page 9**

**Figure S2, page 10**

**Reference, pages 11-13**

| **Table S1: Summary of studies reporting significant associations of miRNAs with lung cancer in Western populations** | | | | | | | | | | | | | | | | | | |
| --- | --- | --- | --- | --- | --- | --- | --- | --- | --- | --- | --- | --- | --- | --- | --- | --- | --- | --- |
| **miRNA** | **Ref.** | | | | | | | | | | | | | | | | | **Number of studies** |
|  | **17** | **8** | **5** | **4** | **16** | **15** | **9** | **14** | **12** | **10** | **7** | **3** | **6** | **13** | **11** | **2** | **1** |  |
| miR-21 | △↑ |  |  | △↑ |  |  |  | ○ |  |  | △↑ |  |  | ○↑ |  | ○ |  | 6 |
| miR-155 |  |  |  |  | ○↑ |  |  |  | ○↑ |  |  |  | ○↓ |  | △↑ |  |  | 4 |
| miR-126 |  |  |  |  |  |  |  | ○ | ○↓ |  |  |  |  | ○↓ |  |  | ○ | 4 |
| miR-486 |  |  |  |  |  |  |  | ○ |  |  |  |  |  | ○↓ |  | ○ | ○ | 4 |
| miR-17 |  |  |  |  |  |  |  | ○ |  |  |  |  | ○↓ |  |  | ○ | ○ | 4 |
| miR-142-3p |  |  |  |  |  |  |  | ○ |  |  |  | ○ |  |  |  | ○ | ○ | 4 |
| miR-25 | △↓ |  |  |  |  | ○↑ |  |  | ○↑ |  |  |  |  |  |  |  |  | 3 |
| miR-15b |  |  |  |  |  |  |  | ○ |  |  |  | ○ |  |  |  | ○ |  | 3 |
| miR-19b | ○↑ |  |  |  |  |  |  | ○ |  |  |  |  |  |  |  | ○ |  | 3 |
| miR-221 |  |  |  |  |  |  |  | ○ |  |  |  |  | ○↓ |  |  | ○ |  | 3 |
| miR-30c |  |  |  |  |  |  |  | ○ |  |  |  |  |  |  |  | ○ | ○ | 3 |
| miR-92a |  |  |  |  |  |  |  | ○ |  |  |  |  |  |  |  | ○ | ○ | 3 |
| miR-106a |  |  |  |  |  |  |  | ○ |  |  |  |  | ○↓ |  |  | ○ |  | 3 |
| miR-140-5p |  |  |  |  |  |  |  | ○ |  |  |  |  |  |  |  | ○ | ○ | 3 |
| miR-30b |  |  |  |  |  |  |  | ○ |  |  |  |  |  |  |  | ○ | ○ | 3 |
| miR-566 |  |  |  |  | ○↓ |  |  |  |  | △↑ |  |  |  |  |  |  | ○ | 3 |
| miR-145 |  |  |  |  |  |  |  | ○ | ○↓ |  |  |  |  |  |  |  |  | 2 |
| miR-182 |  |  |  |  | ○↓ |  |  |  |  |  |  |  |  | △↑ |  |  |  | 2 |
| miR-223 |  |  |  |  |  |  |  |  | ○↑ |  |  |  |  |  |  |  | ○ | 2 |
| miR-148a |  |  |  |  |  |  |  | ○ |  |  |  |  |  |  |  |  | ○ | 2 |
| miR-197 |  |  |  |  |  |  |  | ○ |  |  |  |  |  |  |  | ○ |  | 2 |
| miR-205 | △- |  | ○↑ |  |  |  |  |  |  |  |  |  |  |  |  |  |  | 2 |
| miR-28-3p |  |  |  |  |  |  |  | ○ |  |  |  |  |  |  |  | ○ |  | 2 |
| miR-320 |  |  |  |  |  |  |  | ○ |  |  |  |  |  |  |  | ○ |  | 2 |
| miR-451 |  |  |  |  |  |  |  | ○ |  |  |  |  |  |  |  | ○ |  | 2 |
| let-7a |  |  |  |  |  |  |  |  |  |  |  |  | ○↓ |  |  |  | ○ | 2 |
| let-7b |  |  |  |  | ○↑ |  |  |  |  |  |  |  |  |  |  |  | ○ | 2 |
| miR-140-3p |  |  |  |  |  |  |  | ○ |  |  |  |  |  |  |  | ○ |  | 2 |
| miR-203 |  |  | ○↑ |  | ○↓ |  |  |  |  |  |  |  |  |  |  |  |  | 2 |
| miR-660 |  |  |  |  |  |  |  | ○ |  |  |  |  |  |  |  | ○ |  | 2 |
| miR-152 |  |  |  | △↑ |  |  |  |  |  |  |  |  |  |  |  |  |  | 1 |
| miR-20a |  |  |  |  |  |  |  |  | ○↑ |  |  |  |  |  |  |  |  | 1 |
| miR-24 |  |  |  |  |  |  |  |  | ○↓ |  |  |  |  |  |  |  |  | 1 |
| miR-210 |  |  |  |  |  |  |  |  |  |  |  |  |  | ○↑ |  |  |  | 1 |
| let-7c |  |  |  |  | ○- |  |  |  |  |  |  |  |  |  |  |  |  | 1 |
| miR-142-5p |  |  |  |  |  |  |  |  |  |  |  |  |  |  |  |  | ○ | 1 |
| miR-148b |  |  |  |  |  |  |  |  |  |  |  |  |  |  |  |  | ○ | 1 |
| miR-16 |  |  |  |  |  |  |  | ○ |  |  |  |  |  |  |  |  |  | 1 |
| miR-183 | ○↓ |  |  |  |  |  |  |  |  |  |  |  |  |  |  |  |  | 1 |
| miR-199a-5p |  |  |  |  |  |  |  |  | ○↓ |  |  |  |  |  |  |  |  | 1 |
| miR-22 |  |  |  |  |  |  |  |  |  |  |  |  |  |  |  |  | ○ | 1 |
| miR-29c |  |  |  |  |  |  |  |  |  |  |  |  | ○↑ |  |  |  |  | 1 |
| miR-34b |  |  | ○↑ |  |  |  |  |  |  |  |  |  |  |  |  |  |  | 1 |
| miR-429 |  |  | ○↑ |  |  |  |  |  |  |  |  |  |  |  |  |  |  | 1 |
| let-7d |  |  |  |  |  |  |  |  |  |  |  |  |  |  |  |  | ○ | 1 |

| **Table S1: Continued** | | | | | | | | | | | | | | | | | | |
| --- | --- | --- | --- | --- | --- | --- | --- | --- | --- | --- | --- | --- | --- | --- | --- | --- | --- | --- |
| **miRNA** | **Ref.** | | | | | | | | | | | | | | | | | **Number of studies** |
|  | **17** | **8** | **5** | **4** | **16** | **15** | **9** | **14** | **12** | **10** | **7** | **3** | **6** | **13** | **11** | **2** | **1** |  |
| let-7f-5p |  |  |  |  |  |  |  |  | ○↓ |  |  |  |  |  |  |  |  | 1 |
| miR-101 |  |  |  |  |  |  |  | ○ |  |  |  |  |  |  |  |  |  | 1 |
| miR-103 |  |  |  |  |  |  |  |  |  |  |  |  |  |  |  |  | ○ | 1 |
| miR-10b |  |  |  |  |  |  |  |  |  |  |  |  |  |  | △↑ |  |  | 1 |
| miR-122 |  |  |  |  | ○↓ |  |  |  |  |  |  |  |  |  |  |  |  | 1 |
| miR-1243 |  |  |  |  | ○↓ |  |  |  |  |  |  |  |  |  |  |  |  | 1 |
| miR-125b |  |  | ○↑ |  |  |  |  |  |  |  |  |  |  |  |  |  |  | 1 |
| miR-1267 |  |  |  |  | ○- |  |  |  |  |  |  |  |  |  |  |  |  | 1 |
| miR-133a |  |  |  |  |  |  |  | ○ |  |  |  |  |  |  |  |  |  | 1 |
| miR-133b |  |  |  |  |  |  |  |  |  |  |  |  |  |  |  |  | ○ | 1 |
| miR-139-5p |  |  |  |  |  |  |  |  |  |  |  |  |  |  |  |  | ○ | 1 |
| miR-141 |  |  |  |  |  |  |  |  |  |  |  |  |  |  | △↑ |  |  | 1 |
| miR-146b |  |  |  |  |  |  |  |  |  |  |  |  | ○↓ |  |  |  |  | 1 |
| miR-146b-3p |  |  |  |  |  |  |  |  |  | △↑ |  |  |  |  |  |  |  | 1 |
| miR-152-3p |  |  |  |  |  |  |  |  | ○↓ |  |  |  |  |  |  |  |  | 1 |
| miR-15a |  |  |  |  |  |  |  |  |  |  |  | ○ |  |  |  |  |  | 1 |
| miR-191 |  |  |  |  |  |  |  |  |  |  |  |  |  |  |  |  | ○ | 1 |
| miR-193a-3p |  |  |  |  |  | ○↑ |  |  |  |  |  |  |  |  |  |  |  | 1 |
| miR-193a-5p |  |  |  |  | ○↓ |  |  |  |  |  |  |  |  |  |  |  |  | 1 |
| miR-200b |  |  | ○↑ |  |  |  |  |  |  |  |  |  |  |  |  |  |  | 1 |
| miR-200c |  |  |  |  | ○↑ |  |  |  |  |  |  |  |  |  |  |  |  | 1 |
| miR-206 |  |  |  |  | ○- |  |  |  |  |  |  |  |  |  |  |  |  | 1 |
| miR-214 |  |  |  |  |  | ○↑ |  |  |  |  |  |  |  |  |  |  |  | 1 |
| miR-218 |  |  |  |  | ○↓ |  |  |  |  |  |  |  |  |  |  |  |  | 1 |
| miR-26a |  |  |  |  |  |  |  |  |  |  |  |  |  |  |  |  | ○ | 1 |
| miR-26b |  |  |  |  |  |  |  |  |  |  |  |  |  |  |  |  | ○ | 1 |
| miR-27a |  |  |  |  |  |  |  |  |  |  |  |  | ○↓ |  |  |  |  | 1 |
| miR-27b |  |  |  |  |  |  |  |  |  |  |  | ○ |  |  |  |  |  | 1 |
| miR-28-5p |  |  |  |  |  |  |  |  |  |  |  |  |  |  |  |  | ○ | 1 |
| miR-296-5p |  |  |  |  |  |  |  |  | ○↑ |  |  |  |  |  |  |  |  | 1 |
| miR-29a |  |  |  |  |  |  |  |  |  |  |  |  |  |  |  |  | ○ | 1 |
| miR-301 |  |  |  |  |  |  |  |  |  |  |  | ○ |  |  |  |  |  | 1 |
| miR-30c-1-3p |  |  |  |  |  |  |  |  |  | △↑ |  |  |  |  |  |  |  | 1 |
| miR-32 |  |  |  |  |  |  |  |  |  |  |  |  |  |  |  |  | ○ | 1 |
| miR-328 |  |  |  |  |  |  |  |  |  |  |  |  |  |  |  |  | ○ | 1 |
| miR-331-3p |  |  |  |  |  |  |  |  |  |  |  |  |  |  |  |  | ○ | 1 |
| miR-335-3p |  |  |  |  |  |  |  |  |  |  | △↑ |  |  |  |  |  |  | 1 |
| miR-340-3p |  |  |  |  | ○↑ |  |  |  |  |  |  |  |  |  |  |  |  | 1 |
| miR-342-3p |  |  |  |  |  |  |  |  |  |  |  |  |  |  |  |  | ○ | 1 |
| miR-34a |  |  |  |  |  |  |  |  |  |  |  |  |  |  | △↑ |  |  | 1 |
| miR-3662 |  |  |  |  |  |  | ○↑ |  |  |  |  |  |  |  |  |  |  | 1 |
| miR-374a |  |  |  |  |  |  |  |  |  |  |  |  |  |  |  |  | ○ | 1 |
| miR-376a |  |  |  |  |  |  |  |  |  |  |  |  |  |  |  |  | ○ | 1 |
| miR-411 |  |  |  |  | ○↓ |  |  |  |  |  |  |  |  |  |  |  |  | 1 |
| miR-432-3p |  |  |  |  |  |  |  |  |  |  |  |  |  |  |  |  | ○ | 1 |

| **Table S1: Continued** | | | | | | | | | | | | | | | | | | |
| --- | --- | --- | --- | --- | --- | --- | --- | --- | --- | --- | --- | --- | --- | --- | --- | --- | --- | --- |
| **miRNA** | **Ref.** | | | | | | | | | | | | | | | | | **Number of studies** |
|  | **17** | **8** | **5** | **4** | **16** | **15** | **9** | **14** | **12** | **10** | **7** | **3** | **6** | **13** | **11** | **2** | **1** |  |
| miR-4478 |  | ○↑ |  |  |  |  |  |  |  |  |  |  |  |  |  |  |  | 1 |
| miR-448 |  | ○↑ |  |  |  |  |  |  |  |  |  |  |  |  |  |  |  | 1 |
| miR-450b-5p |  |  |  |  | ○↓ |  |  |  |  |  |  |  |  |  |  |  |  | 1 |
| miR-483-5p |  |  |  |  |  | ○↑ |  |  |  |  |  |  |  |  |  |  |  | 1 |
| miR-484 |  |  |  |  |  |  |  |  |  |  |  |  |  |  |  |  | ○ | 1 |
| miR-485-3p |  |  |  |  | ○↓ |  |  |  |  |  |  |  |  |  |  |  |  | 1 |
| miR-517b |  |  |  |  | ○↓ |  |  |  |  |  |  |  |  |  |  |  |  | 1 |
| miR-519a |  |  |  |  | ○- |  |  |  |  |  |  |  |  |  |  |  |  | 1 |
| miR-520f |  |  |  |  | ○- |  |  |  |  |  |  |  |  |  |  |  |  | 1 |
| miR-543 |  |  |  |  | ○- |  |  |  |  |  |  |  |  |  |  |  |  | 1 |
| miR-550 |  |  |  |  |  |  |  |  |  | △↑ |  |  |  |  |  |  |  | 1 |
| miR-616-5p |  |  |  |  |  |  |  |  |  | △↑ |  |  |  |  |  |  |  | 1 |
| miR-642 |  |  |  |  | ○↓ |  |  |  |  |  |  |  |  |  |  |  |  | 1 |
| miR-661 |  |  |  |  | ○↓ |  |  |  |  |  |  |  |  |  |  |  |  | 1 |
| miR-7 |  |  |  |  |  | ○↑ |  |  |  |  |  |  |  |  |  |  |  | 1 |
| miR-720 |  |  |  |  | ○- |  |  |  |  |  |  |  |  |  |  |  |  | 1 |
| miR-939 |  |  |  |  |  |  |  |  |  | △↑ |  |  |  |  |  |  |  | 1 |
| miR-944 |  |  |  |  |  |  | ○↑ |  |  |  |  |  |  |  |  |  |  | 1 |
| miR-98 |  |  |  |  |  |  |  |  |  |  |  |  |  |  |  |  | ○ | 1 |

○ represents miRNAs which are part of a panel; △ represents miRNAs which have only been analyzed individually and not as part of a miRNA panel; ↑ represents up-regulation; ↓ represents down-regulation; - represents no difference in overall study population.

| **Table S2: Diagnostic performance of miRNAs and miRNA panels in lung cancer stage subgroup in Western populations** | | | | | | | | | | | |
| --- | --- | --- | --- | --- | --- | --- | --- | --- | --- | --- | --- |
| **Study** | **Country** | **Cases vs, Controls** | | | **Specimen** | **miRNA** | **SEN** | **SPE** | **AUC** | **Histology** | **Stage** |
|  |  | **Number** | **Age(y)** | **Male (%)** |  |  |  |  |  |  |  |
| Powrozek, 2016 [8] | Poland | 90/85 | 64/57 | 69/60 | plasma | miR-448, miR-4478 | 89 | 79 | 0.90 | Any LC | I-IV |
|  |  | 40/85 | NA/57 | NA/60 |  |  | 90 | 76 | 0.90 | NSCLC | IA-IIB |
| Halvorsen, 2016 [5] | Norway | 100/58 | 63/58 | 72/59 | serum | miR-429, miR-205, miR-200b, miR-203, miR-125b, miR-34b | **88** | **71** | **0.89** | NSCLC | I-IV |
|  |  | 79/58 | NA/58 | NA/59 |  |  | **85** | **74** | **0.88** | NSCLC | IA-IIB |
| Wozniak, 2015 [16] | Russia | 100/100 | 63/60 | 86/71 | plasma | let-7c, miR-122, miR-182, miR-193a-5p, miR-200c, miR-203, miR-218, miR-155, let-7b, miR-411, miR-450b-5p, miR-485-3p, miR-519a, miR-642, miR-517b, miR-520f, miR-206, miR-566, miR-661, miR-340*, miR-1243, miR-720, miR-543, miR-1267 | 83 | 80 | 0.92 | NSCLC | IA-IIIA |
|  |  | 49/100 | NA/60 | NA/71 |  |  | / | / | 0.96 | NSCLC | I |
|  |  | 21/100 | NA/60 | NA/71 |  |  | / | / | 0.98 | NSCLC | II |
|  |  | 30/100 | NA/60 | NA/71 |  |  | / | / | 0.97 | NSCLC | III |
| Powrozek, 2015 [9] | Poland | 90/85 | 65/57 | 62/60 | plasma | miR-944 | 82 | 91 | 0.91 | Any LC | I-IV |
|  |  |  |  |  |  | miR-3662 | 72 | 94 | 0.90 |  |  |
|  |  | 40/85 | NA/57 | NA/60 |  | miR-944 | 80 | 90 | 0.90 | NSCLC | I-IIIA |
|  |  |  |  |  |  | miR-3662 | 60 | 94 | 0.85 |  |  |
|  |  | 90/85 | 65/57 | 62/60 |  | miR-944, miR-3662 | 82 | 92 | 0.91 | Any LC | I-IV |
|  |  | 40/85 | NA/57 | NA/60 |  |  | 92 | 86 | 0.88 | NSCLC | I-IIIA |
| Shen, 2011 [13] | USA | 58/29 | 68/66 | 66/66 | plasma | miR-21, miR-486-5p, miR-126, miR-210 | 86 | 97 | 0.93 | NSCLC | I-IV |
|  |  | 15/29 | NA/66 | NA/66 |  |  | 73 | 97 | / | NSCLC | I |
|  |  | 15/29 | NA/66 | NA/66 |  |  | 87 | 97 | / | NSCLC | II |
|  |  | 12/29 | NA/66 | NA/66 |  |  | 92 | 97 | / | NSCLC | III |
|  |  | 16/29 | NA/66 | NA/66 |  |  | 94 | 97 | / | NSCLC | IV |
| Bianchi, 2011 [1] | Italy | 34/30 | 62/59 | 68/67 | serum | miR-92a, miR-486-5p, miR-484, miR-191, miR-26a, let-7b, miR-328, miR-30c, miR-342-3p, miR-30b, miR-26b, miR-142-3p, miR-331-3p, miR-103, miR-17, let-7a, miR-126, miR-22, miR-374a, miR-148b, let-7d, miR-28-5p, miR-139-5p, miR-376a, miR-98, miR-223, miR-142-5p, miR-140-5p, miR-29a, miR-148a, miR-133b, miR-32, miR-566, miR-432* | **71** | **90** | **0.89** | NSCLC | I-IV |
|  |  | 22/30 | 62/59 | 73/67 |  |  | **59** | **90** | **0.89** | NSCLC | I |
|  |  | 12/30 | 60/59 | 58/67 |  |  | **92** | **90** | **0.88** | NSCLC | II-IV |

SENs, SPEs and AUCs in bold fonts represent results from validation set (non-bold fonts represent results without validation).

Abbreviations: SEN: sensitivity; SPE: specificity; AUC: area under the curve; LC: lung cancer; NSCLC: non-small cell lung cancer; ADC: adenocarcinoma; SCC: squamous cell carcinoma; NA: not available.

| **Table S3: Opposite regulated miRNAs in lung cancer blood samples** | | | | | | | | | | | |
| --- | --- | --- | --- | --- | --- | --- | --- | --- | --- | --- | --- |
| **miRNA** | **Ref.** | **First Author, Year** | **Country** | **Number** | **histology** | **stage** | **specimen** | **Centrifugation** | **Extraction** | **Normalization** |  |
| miR-155 ↑ | 16 | Wozniak, 2015 | Russia | 100/100 | NSCLC | IA-IIIA | plasma | 2000g for 10min | NucleoSpin miRNA Plasma kit | U6 snRNA, ath-miR-159a |  |
| miR-155 ↑ | 12 | Sanfiorenzo, 2013 | France | 52/20 | NSCLC | IA-IIIA | plasma | 3000rpm for 10min | miRNeasy Mini Kit | miR-192, miR-16 |  |
| miR-155 ↑ | 11 | Roth, 2011 | Germany | 35/28 | LC | I-IV | serum | repeatedly centrifuged | mirVana PARIS Kit | miR­16 |  |
| miR-155 ↓ | 6 | Heegaard, 2012 | USA | 99/220 | ADC | IA-IIB | serum | NA | Total RNA purification kit | plate regression |  |
| **miR-25 ↑** | 15 | Wang, 2015 | USA | 108/48 | NSCLC | I-IV | serum | 1500gfor 10min | TRIzol Reagent | let-7d/g/i trio |  |
| miR-25 ↑ | 12 | Sanfiorenzo, 2013 | France | 52/20 | NSCLC | IA-IIIA | plasma | 3000rpm for 10min | miRNeasy Mini Kit | miR-192, miR-16 |  |
| miR-25 ↓ | 17 | Zaporozhchenko, 2016 | Russia | 75/50 | LC | IIA-IV | plasma | [400g for 20min, 800g for 20min], thaw[3000g for 5min] | single-phase phenol-free extraction | miR-16 |  |
| miR-182 ↑ | 13 | Shen, 2011 | USA | 58/29 | NSCLC | I-IV | plasma | 1300g for 10min | mirVana miRNA Isolation Kit | miR­16 |  |
| miR-182 ↓ | 16 | Wozniak, 2015 | Russia | 100/100 | NSCLC | IA-IIIA | plasma | 2000g for 10min | NucleoSpin miRNA Plasma kit | U6 snRNA, ath-miR-159a |  |
| **miR-205 ↑** | 5 | Halvorsen, 2016 | Norway | 100/58 | NSCLC | I-IV | serum | 1811g for 10min | miRCURY RNA isolation kit | miR-220, miR-19b, U6 snRNA |  |
| miR-205 - | 17 | Zaporozhchenko, 2016 | Russia | 75/50 | LC | IIA-IV | plasma | [400g for 20min, 800g for 20min], thaw[3000g for 5min] | single-phase phenol-free extraction | miR-16 |  |
| miR-566 ↑ | 10 | Rani, 2013 | Ireland | 40/40 | ADC | I-IV | serum | NA | TriReagent | mean C_T_ |  |
| miR-566 ↓ | 16 | Wozniak, 2015 | Russia | 100/100 | NSCLC | IA-IIIA | plasma | 2000g for 10min | NucleoSpin miRNA Plasma kit | U6 snRNA, ath-miR-159a |  |
| **miR-203 ↑** | 5 | Halvorsen, 2016 | Norway | 100/58 | NSCLC | I-IV | serum | 1811g for 10min | miRCURY RNA isolation kit | miR-220, miR-19b, U6 snRNA |  |
| miR-203 ↓ | 16 | Wozniak, 2015 | Russia | 100/100 | NSCLC | IA-IIIA | plasma | 2000g for 10min | NucleoSpin miRNA Plasma kit | U6 snRNA, ath-miR-159a |  |

↑ represents up-regulation; ↓ represents down-regulation; - represents no difference in overall study population; bold fonts represent results from validation set (non-bold fonts represent results without validation); NA: not available.

| **Table S4: Protocols of blood miRNA detection** | | | | |
| --- | --- | --- | --- | --- |
| **Ref.** | **Specimen** | **Centrifugation** | **Extraction** | **Normalization** |
| 17 | plasma | 400g for 20min, 800g for 20min, [thaw]3000g for 5min | single-phase phenol-free extraction | miR-16 |
| 8 | plasma | 3000rpm for 10min | miRNeasy serum/plasma kit | U6 snRNA |
| 5 | serum | 1811g for 10min | miRCURY RNA isolation kit | miR-220, miR-19b, U6 snRNA |
| 4 | plasma | 4000rpm for 10min, 12000rpm for 15min | miR-Neasy Mini kit | cel-miR-39 |
| 16 | plasma | 2000g for 10min | NucleoSpin miRNA Plasma kit | U6 snRNA, ath-miR-159a |
| 15 | serum | 1500g for 10min | TRIzol Reagent | let-7d/g/i trio |
| 9 | plasma | 1200g for 12min | miRNeasy serum and plasma kit | U6 snRNA |
| 14 | plasma | 1258g | mirVana PARIS kit | U6 snRNA |
| 15 | plasma | 3000rpm for 10min | miRNeasy Mini Kit | miR-192, miR-16 |
| 10 | serum | NA | TriReagent | mean C_T_ |
| 7 | plasma | 1300g for 10min | mirVana PARIS kit | copy numbers |
| 3 | serum | NA | mirVana PARIS Kit | ΔCt value matrix |
| 6 | serum | NA | Total RNA purification kit | plate regression |
| 13 | plasma | 1300g for 10min | mirVana miRNA Isolation Kit | miR­16 |
| 11 | serum | repeatedly centrifuged | mirVana PARIS Kit | miR­16 |
| 2 | plasma | 1258g | mirVana PARISKit | U6 snRNA |
| 1 | serum | 3000rpm (1000-1300g) for 10min | Trizol LS combined with mirVana Kit | HK normalization, median normalization |

NA: not available.


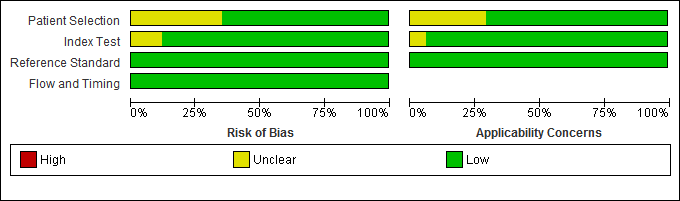


**Figure S1**: Risk of bias and applicability concerns graph: review authors' judgements about each domain presented as percentages across included studies.


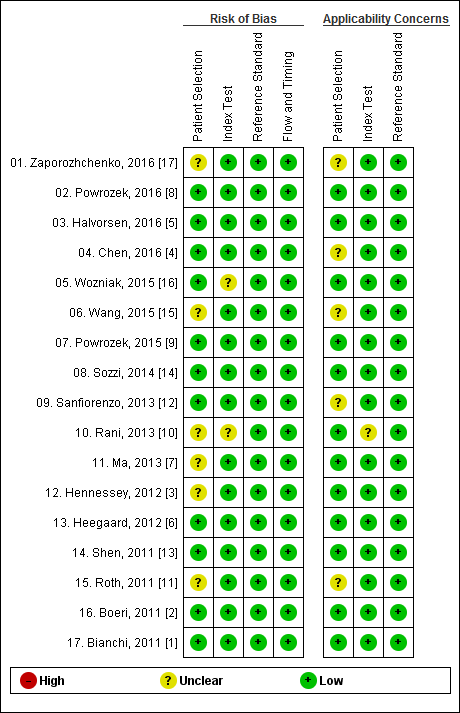


**Figure S2**: Risk of bias and applicability concerns summary: review authors' judgements about each domain for each included study.

**REFERENCES**

1. Bianchi F, Nicassio F, Marzi M, Belloni E, Dall'olio V, Bernard L, Pelosi G, Maisonneuve P, Veronesi G, Di Fiore PP. A serum circulating miRNA diagnostic test to identify asymptomatic high-risk individuals with early stage lung cancer. *EMBO Mol Med* 2011;**3**: 495-503.

2. Boeri M, Verri C, Conte D, Roz L, Modena P, Facchinetti F, Calabro E, Croce CM, Pastorino U, Sozzi G. MicroRNA signatures in tissues and plasma predict development and prognosis of computed tomography detected lung cancer. *Proc Natl Acad Sci U S A* 2011;**108**: 3713-8.

3. Hennessey PT, Sanford T, Choudhary A, Mydlarz WW, Brown D, Adai AT, Ochs MF, Ahrendt SA, Mambo E, Califano JA. Serum microRNA biomarkers for detection of non-small cell lung cancer. *PLoS One* 2012;**7**: e32307.

4. Chen H, Liu H, Zou H, Chen R, Dou Y, Sheng S, Dai S, Ai J, Melson J, Kittles RA, Pirooznia M, Liptay MJ, et al. Evaluation of Plasma miR-21 and miR-152 as Diagnostic Biomarkers for Common Types of Human Cancers. *J Cancer* 2016;**7**: 490-9.

5. Halvorsen AR, Bjaanaes M, LeBlanc M, Holm AM, Bolstad N, Rubio L, Penalver JC, Cervera J, Mojarrieta JC, Lopez-Guerrero JA, Brustugun OT, Helland A. A unique set of 6 circulating microRNAs for early detection of non-small cell lung cancer. *Oncotarget* 2016;**7**: 37250-9.

6. Heegaard NH, Schetter AJ, Welsh JA, Yoneda M, Bowman ED, Harris CC. Circulating micro-RNA expression profiles in early stage nonsmall cell lung cancer. *Int J Cancer* 2012;**130**: 1378-86.

7. Ma J, Li N, Guarnera M, Jiang F. Quantification of Plasma miRNAs by Digital PCR for Cancer Diagnosis. *Biomarker insights* 2013;**8**: 127-36.

8. Powrozek T, Krawczyk P, Kowalski DM, Kuznar-Kaminska B, Winiarczyk K, Olszyna-Serementa M, Batura-Gabryel H, Milanowski J. Application of plasma circulating microRNA-448, 506, 4316, and 4478 analysis for non-invasive diagnosis of lung cancer. *Tumour biology : the journal of the International Society for Oncodevelopmental Biology and Medicine* 2016;**37**: 2049-55.

9. Powrozek T, Krawczyk P, Kowalski DM, Winiarczyk K, Olszyna-Serementa M, Milanowski J. Plasma circulating microRNA-944 and microRNA-3662 as potential histologic type-specific early lung cancer biomarkers. *Translational research : the journal of laboratory and clinical medicine* 2015;**166**: 315-23.

10. Rani S, Gately K, Crown J, O'Byrne K, O'Driscoll L. Global analysis of serum microRNAs as potential biomarkers for lung adenocarcinoma. *Cancer Biol Ther* 2013;**14**: 1104-12.

11. Roth C, Kasimir-Bauer S, Pantel K, Schwarzenbach H. Screening for circulating nucleic acids and caspase activity in the peripheral blood as potential diagnostic tools in lung cancer. *Mol Oncol* 2011;**5**: 281-91.

12. Sanfiorenzo C, Ilie MI, Belaid A, Barlesi F, Mouroux J, Marquette CH, Brest P, Hofman P. Two panels of plasma microRNAs as non-invasive biomarkers for prediction of recurrence in resectable NSCLC. *PLoS One* 2013;**8**: e54596.

13. Shen J, Todd NW, Zhang H, Yu L, Lingxiao X, Mei Y, Guarnera M, Liao J, Chou A, Lu CL, Jiang Z, Fang H, et al. Plasma microRNAs as potential biomarkers for non-small-cell lung cancer. *Laboratory investigation; a journal of technical methods and pathology* 2011;**91**: 579-87.

14. Sozzi G, Boeri M, Rossi M, Verri C, Suatoni P, Bravi F, Roz L, Conte D, Grassi M, Sverzellati N, Marchiano A, Negri E, et al. Clinical utility of a plasma-based miRNA signature classifier within computed tomography lung cancer screening: a correlative MILD trial study. *Journal of clinical oncology : official journal of the American Society of Clinical Oncology* 2014;**32**: 768-73.

15. Wang C, Ding M, Xia M, Chen S, Van Le A, Soto-Gil R, Shen Y, Wang N, Wang J, Gu W, Wang X, Zhang Y, et al. A Five-miRNA Panel Identified From a Multicentric Case-control Study Serves as a Novel Diagnostic Tool for Ethnically Diverse Non-small-cell Lung Cancer Patients. *EBioMedicine* 2015;**2**: 1377-85.

16. Wozniak MB, Scelo G, Muller DC, Mukeria A, Zaridze D, Brennan P. Circulating MicroRNAs as Non-Invasive Biomarkers for Early Detection of Non-Small-Cell Lung Cancer. *PLoS One* 2015;**10**: e0125026.

17. Zaporozhchenko IA, Morozkin ES, Skvortsova TE, Ponomaryova AA, Rykova EY, Cherdyntseva NV, Polovnikov ES, Pashkovskaya OA, Pokushalov EA, Vlassov VV, Laktionov PP. Plasma miR-19b and miR-183 as Potential Biomarkers of Lung Cancer. *PLoS One* 2016;**11**: e0165261.
